# Supplementary material for: Anterograde trafficking of Toll‐like receptors requires the cargo sorting adaptors TMED‐2 and 7
Source: Traffic. 2023 Jul 26;24(11):508–21. doi: 10.1111/tra.12912 (PMC10946956; doi:10.1111/tra.12912)
Supplement: Supplementary file 1 — Figure S1: Mass photometry of TMED2/7 oligomers. TMED2 and 7 were co‐expressed in a pETDUet vector with Strep and FLAG affinity tags respectively using T7 Shuffle‐PDI Escherichia Coli (E. coli) cells. Soluble lysate was purified by Strep affinity chromatography and S75 gel filtration. An included peak (B) from the gel filtration was analysed by mass photometry (see1 for the method). The presence of both TMED2 and 7 was verified by western blot. Figure S2: Removal of FF‐motif retains both TMED2 and TMED7 in the ER and affects the ER Export of some TLRs in murine BMDM cells. Dual‐colour imaging of murine BMDM cells transduced with lentivirus encoding, (A) TMED7‐ and TMED7T‐HA (top), and TMED2‐ and TMED2T‐HA (bottom); (B) TMED7‐HA (top left) and TMED7T‐HA (top right) with TLR4‐citrine; TMED7‐HA (bottom left) and TMED7T‐HA (bottom right) with TLR3‐citrine; (C) TMED2‐HA (top left) and TMED2T‐HA (top right) with TLR4‐citrine; TMED2‐HA (bottom left) and TMED2T‐HA (bottom right) with TLR3‐citrine. HA‐tag constructs were immunostained and visualised in the far‐red (635 nm) channel and TLRs in the yellow (515 nm) channel, while the ER was immunostained and visualised in the blue (405 nm) channel. The overlay is a merge of the red channel with either the far‐red or the blue channel respectively. Quantification of the Pearson's coefficient (R) for the dual colour merged imaged. Images are representatives of three independent experiments. Scale bar 5–10 μm. [file TRA-24-508-s001.docx]

**SUPPLEMENTARY MATERIAL**


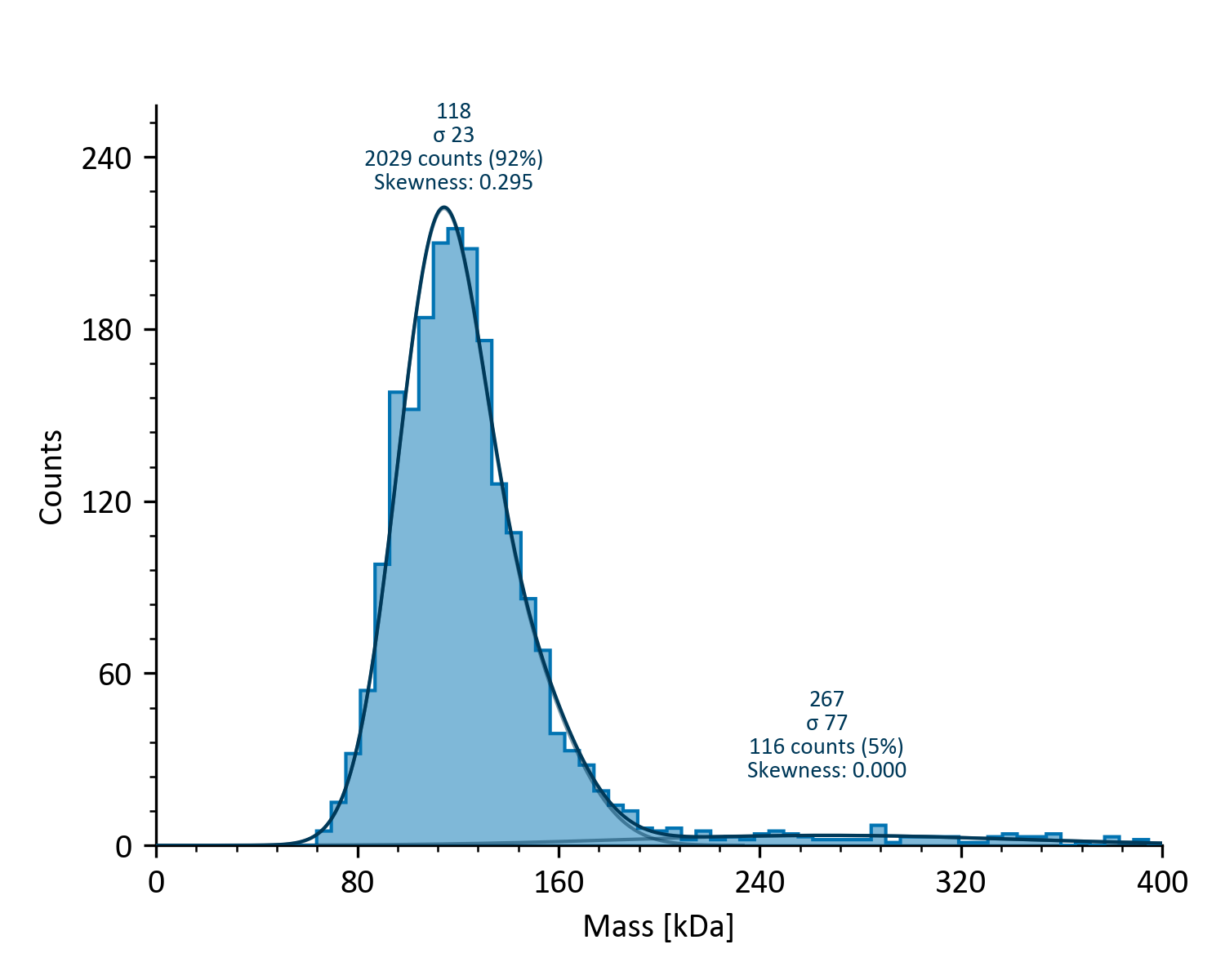


TMED2TMED7 Gel filtration Peak B

**Figure S1: Mass photometry of TMED2/7 oligomers**. TMED2 and 7 were co-expressed in a pETDUet vector with Strep and FLAG affinity tags respectively using T7 Shuffle-PDI *E. coli* cells. Soluble lysate was purified by Strep affinity chromatography and S75 gel filtration. An included peak (B) from the gel filtration was analysed by mass photometry (see^1^ for the method). The presence of both TMED2 and 7 was verified by western blot.

**Figure S2**: **Removal of FF-motif retains both TMED2 and TMED7 in the ER** **and affects the ER Export of some TLRs in murine BMDM cells.** Dual-colour imaging of murine BMDM cells transduced with lentivirus encoding, **A**: TMED7- and TMED7T-HA (top), and TMED2- and TMED2T-HA (bottom); **B**: TMED7-HA (top left) and TMED7T-HA (top right) with TLR4-citrine; TMED7-HA (bottom left) and TMED7T-HA (bottom right) with TLR3-citrine; **C**: TMED2-HA (top left) and TMED2T-HA (top right) with TLR4-citrine; TMED2-HA (bottom left) and TMED2T-HA (bottom right) with TLR3-citrine. HA-tag constructs were immunostained and visualised in the far-red (635 nm) channel and TLRs in the yellow (515 nm) channel, while the ER was immunostained and visualised in the blue (405 nm) channel. The overlay is a merge of the red channel with either the far-red or the blue channel respectively. Quantification of the Pearson’s coefficient (R) for the dual colour merged imaged. Images are representatives of three independent experiments. Scale bar 5 to 10 μm.

1. Saucereau, Y. *et al.* Structure and dynamics of Toll immunoreceptor activation in the mosquito Aedes aegypti. *Nat Commun* **13**, 5110 (2022).
